# Predictors of COVID-19 vaccination status: a nationwide linkage study in Czechia

**DOI:** 10.1093/pubmed/fdag043

**Published:** 2026-06-16

**Authors:** Dagmar Dzúrová, Klára Benešová, Josef Bernard, Klára Hulíková Tesárková, Jiří Jarkovský, Lukáš Kahoun, Gabriela Kšiňanová, Jan Mužík, Pavlína Netrdová, Daniel Szabo, Martin Bobák

**Affiliations:** Department of Social Geography and Regional Development, Faculty of Science, Charles University, Albertov 6, 12800 Prague 2, Czechia; Institute of Health Information and Statistics, Palackého náměstí 4, 12800 Prague 2, Czechia; Institute of Biostatistics and Analyses, Faculty of Medicine, Masaryk University, Kamenice 126, 62500 Brno, Czechia; Institute of Sociology, Czech Academy of Sciences, Jilská 1, 11000 Prague 1, Czechia; Department of Demography and Geodemography, Faculty of Science, Charles University, Albertov 6, 12800 Prague 2, Czechia; Institute of Health Information and Statistics, Palackého náměstí 4, 12800 Prague 2, Czechia; Institute of Biostatistics and Analyses, Faculty of Medicine, Masaryk University, Kamenice 126, 62500 Brno, Czechia; Institute of Health Information and Statistics, Palackého náměstí 4, 12800 Prague 2, Czechia; Department of Demography and Geodemography, Faculty of Science, Charles University, Albertov 6, 12800 Prague 2, Czechia; RECETOX, Faculty of Science, Masaryk University, Kamenice 753/5, 62500 Brno, Czechia; Institute of Health Information and Statistics, Palackého náměstí 4, 12800 Prague 2, Czechia; Institute of Biostatistics and Analyses, Faculty of Medicine, Masaryk University, Kamenice 126, 62500 Brno, Czechia; Department of Social Geography and Regional Development, Faculty of Science, Charles University, Albertov 6, 12800 Prague 2, Czechia; RECETOX, Faculty of Science, Masaryk University, Kamenice 753/5, 62500 Brno, Czechia; RECETOX, Faculty of Science, Masaryk University, Kamenice 753/5, 62500 Brno, Czechia; Institute of Epidemiology and Health Care, University College London, 1-19 Torrington Place, London WC1E 6BT, UK

**Keywords:** social inequality, national registers, data linkage, COVID-19, vaccination, Czechia

## Abstract

**Objectives:**

The evidence on coronavirus disease 2019 (COVID-19) vaccine uptake in Central and Eastern Europe remains sparse. We examined predictors of COVID-19 vaccination status in Czechia, using linkage of multiple routine nationwide datasets on objective measures of both vaccination and its predictors.

**Methods:**

We used linked administrative data on 6 398 768 individuals aged 26 and older alive on 31 December 2023. Individual-level data were linked across national registries from the Infectious Diseases Information System, National Health Information System, the National Registry of Reimbursed Health Services, and the Ministry of Labour and Social Affairs.

**Results:**

The prevalence of unvaccinated status was 22.4%; after multivariable adjustment it was associated with all examined characteristics (age, sex, comorbidity index, registration with a general practitioner (GP), socioeconomic status, unemployment, and district of residence). The prevalence of unvaccinated status among individuals defined by the lowest quintile of income receiving social benefits was 42.1%, and the multivariable adjusted odds ratio (OR) was 3.91 (95% CI 3.87–3.96) compared to the highest quintile; the adjusted OR among unemployed vs. employed subjects was 1.52 (95% CI 1.51–1.53). Higher morbidity index and being registered with a GP were associated with lower odds of unvaccinated status.

**Conclusions:**

These results suggest substantial socioeconomic inequalities in objectively recorded vaccination status.

## Introduction

Previous studies of COVID-19 vaccine acceptance and uptake showed associations between vaccination status and sociodemographic characteristics. Lower socioeconomic status (SES), operationalized as lower income, lower educational level, unemployment, and receipt of social benefits, was associated with lower COVID-19 vaccine acceptance and uptake in studies based on medical registry data from the USA,^1^ Belgium,^2^ Switzerland,^3^ the Netherlands,^4^ and Sweden.^5^ An association of income and education with COVID-19 vaccine acceptance and uptake was reported in a survey in 13 countries^6^ and in several review papers.^7^^,^^8^ In addition, younger individuals were less likely to plan to get vaccinated or to receive the vaccine, including a booster.^1^^,^^4^^,^^5^^,^^7^ Several studies showed an association of prior individual health status and behaviors with the COVID-19 vaccine uptake.^1^^,^^3^

The vast majority of existing evidence comes from Western Europe or North America, while in Central and Eastern Europe (CEE), the evidence remains sparse, with reports generally confirming lower vaccination willingness and coverage among disadvantaged socioeconomic groups.^9–15^ Virtually all previous studies in CEE relied on self-report; no study from CEE utilized a dataset that combines national registers with officially recorded COVID-19 vaccination status.

Filling the knowledge gap about vaccination acceptance is important, given the concerns about future pandemic(s). This study aims to provide evidence from the CEE region by linking several Czech national registers to investigate the associations between COVID-19 vaccination status and sociodemographic factors, income, unemployment, and prior health to improve identification of population groups ineffectively addressed by the COVID-19 vaccination efforts.

## Methods

### Study design, data sources, study, and study population

This retrospective cohort study used administrative data covering all individuals living in Czechia (population 10 million). The baseline population consisted of 7 610 761 individuals aged 26 and older (since data on income are unavailable for younger persons), alive as of 31 December 2023, and registered in the National Health Information System (NHIS) before 2021. As a validation that a person actually lived in Czechia, we only included persons who received any care covered by health insurance and/or were registered for primary care in 2022 and 2023.

**Figure 1:**
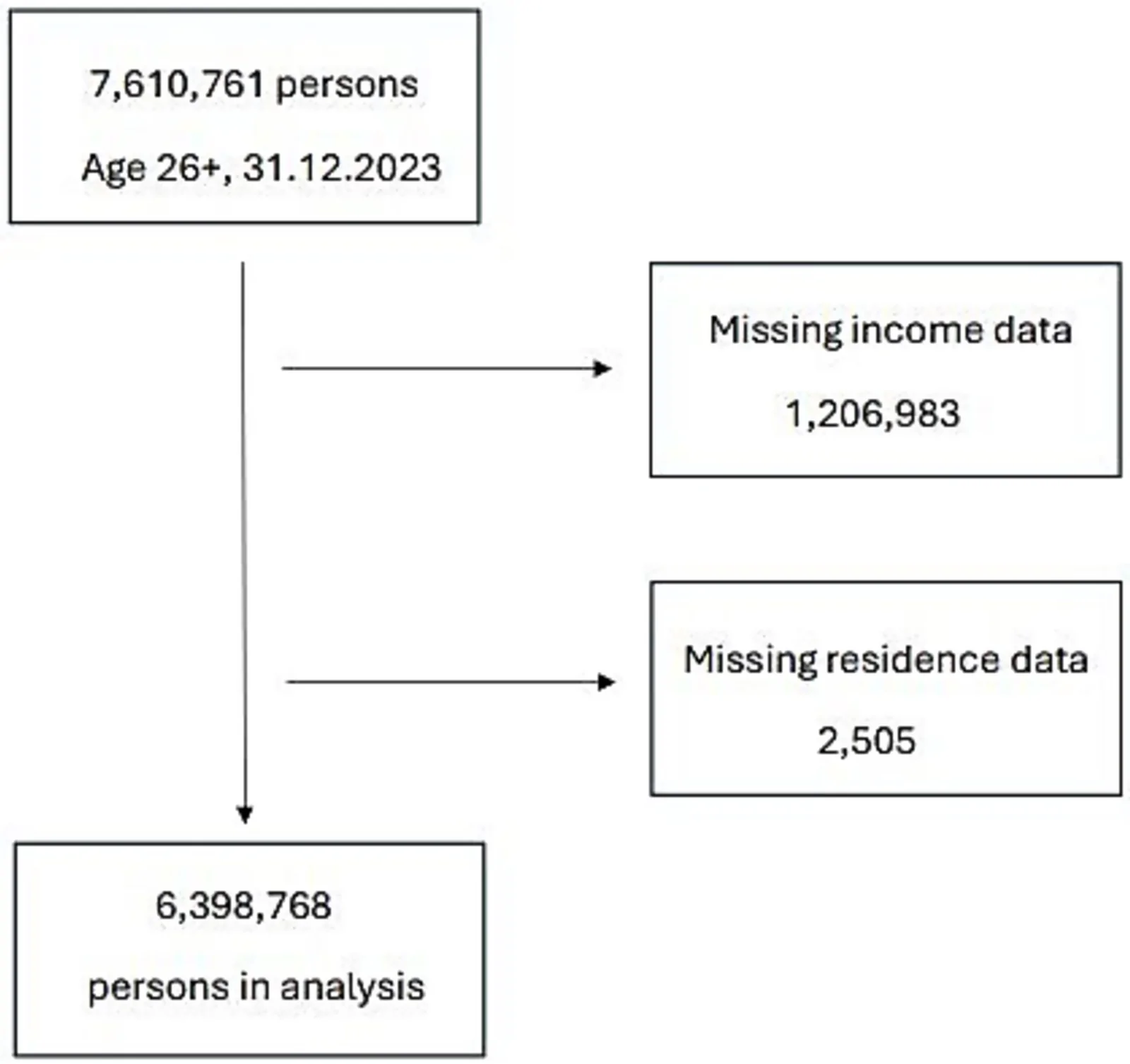
Flow chart of data linkage and exclusions due to missingness.

Individual-level data were linked across the following national registers: the Infectious Diseases Information System (ISID), the National Registry of Reimbursed Health Services (NRRHS), and the Ministry of Labor and Social Affairs database. The datasets were maintained, checked for accuracy, linked, and analyzed by the Institute of Health Information and Statistics of the Czech Republic (IHIS CR). Individuals with incomplete data were excluded due to missing data on income and/or place, or residence, leaving 6 398 768 persons in the analysis as the study population (Fig. 1). The comparison of the analytical sample with persons with missing data suggested lower vaccine uptake in excluded subjects. The excluded subjects were younger on average, with fewer comorbidities, and two-thirds were men (Supplementary Table 1). The main reason for exclusion was missing data on income (reducing the analytical sample by 16%), because taxable income information is not recorded for students, self-employed, or long-term unemployed persons. Unfortunately, the administrative dataset does not contain sufficient information to plausibly impute income in these groups.

**Figure 2:**
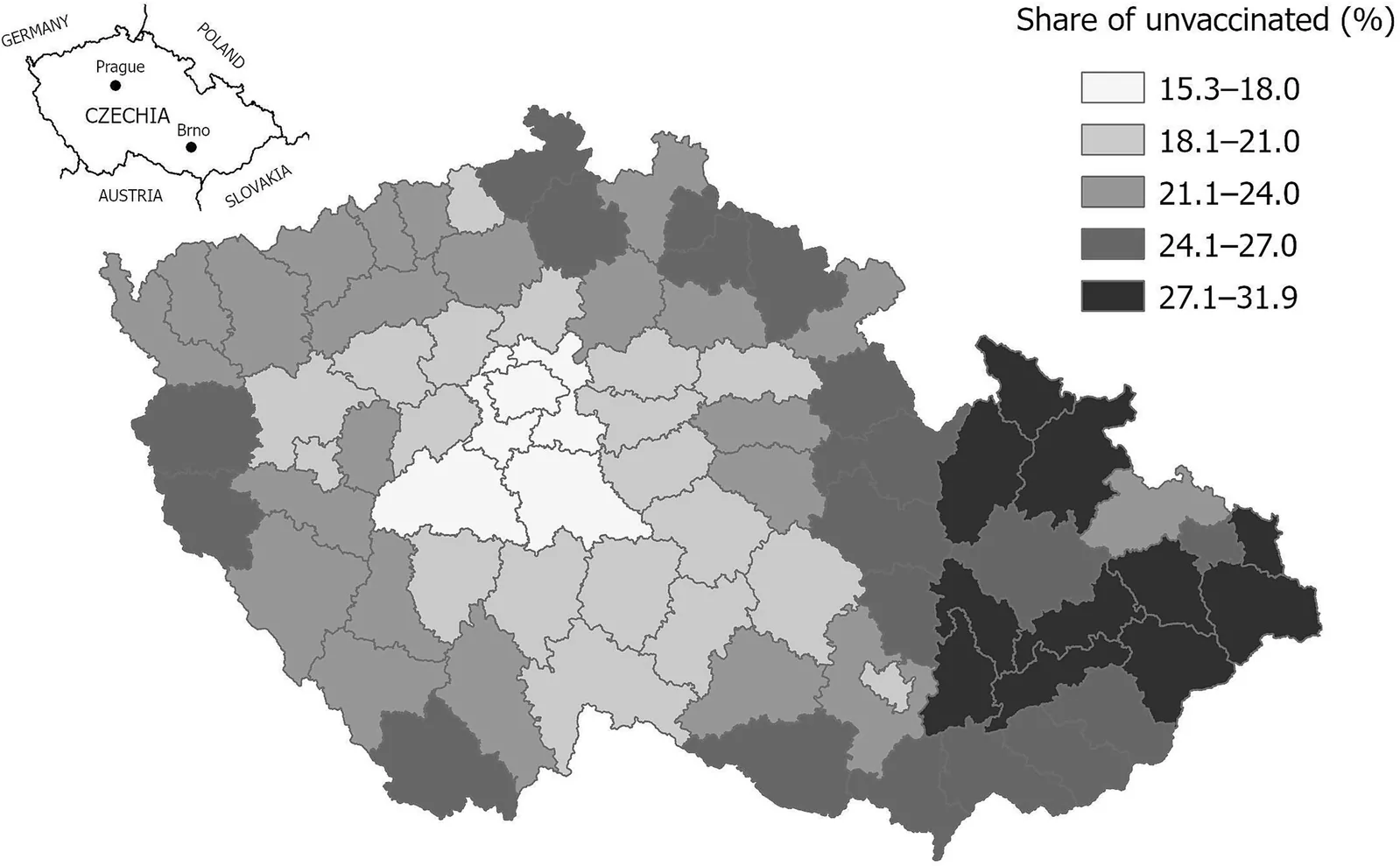
Geographical distribution of COVID-19 unvaccinated status by 77 administrative districts of Czechia, as of 31 December 2023 (%).

### Dependent variable

The study outcome was COVID-19 vaccination status recorded by NHIS until the end of 2023, which was dichotomized as complete primary vaccination (two doses of mRNA or AstraZeneca vaccines or one dose of the Johnson & Johnson vaccine), regardless of a booster dose vs. individuals who were either unvaccinated or had incomplete primary vaccination.

### Independent variables

The NHIS includes *demographic characteristics*, such as age, sex, and place of permanent residence at the end of 2023. The level of the district (*N* = 77) was used in these analyses as a covariate. District IDs were included in the model as fixed variables, thus controlling for different levels of vaccination coverage in individual regions. Prague, which had the highest level of vaccination coverage, was chosen as the reference category. In addition, the following variables were used.

SES was assessed using two dimensions derived from the database of the Ministry of Labor and Social Affairs. The first is based on income, estimated from annual assessments based on gross salary (except self-employed persons) and old-age pensions. For economically active persons, income subject to social insurance payment was used as an input variable as the average monthly income for each year, 2019–23; this was converted into an annual income percentile, from which a median was calculated for the 2019–23 period. This procedure smooths random annual fluctuations in income while also allowing the indicator value to be obtained for persons who had no income in any year. For persons receiving old-age pensions, the index value was derived from the pension amount, which reflects the amount of income before retirement age. The amount of retirement pensions for 2023 was converted into percentiles. For both economically active and retired persons, the estimated income was categorized into quintiles. For a finer classification of persons at the very bottom of the income distribution, the lowest quintile was further divided into two parts, with a sixth group consisting of persons who received one of the social benefits intended for persons in a difficult social situation for at least six months in 2019–23. This formed six categories of the variable SES.

In addition to income-based SES, *unemployment* was included as a dichotomous variable. The ‘exposed’ category comprised individuals who experienced unemployment, defined as being registered with the Labor Office for at least 6 months between 2019 and 2023.

As sensitivity checks, we conducted separate analyses of economically active individuals vs. retirees, and we also compared the main results for economically active subjects with a model in which the categorized SES was replaced by logarithmic transformed (using log base 10) income and a separate variable indicating receipt of social benefits.

Two *health-related covariates* were used. First, a binary indicator of whether the individual was registered with a general practitioner (GP) of January 2021, as a patient’s interaction with their doctor can significantly influence their attitude toward vaccination (Registration with GP: yes/no). Second, we included the variable comorbidity because underlying health conditions are important for vaccination. The multimorbidity was assessed using the Deyo-Charlson Comorbidity Index (DCCI), which summarizes recorded health care provision concerning 17 selected diagnoses over 5 years (2017–21) based on the NRRHS. The *morbidity index* ranged from 0 to 18 in our sample (out of a possible maximum of 29, depending on the severity of different health conditions). As higher values were relatively rare (Table 1), the highest category was defined as DCCI equal to three or more comorbidities. The total score reflects the overall severity of registered comorbidity, with 0 indicating no comorbidity and higher scores representing greater multimorbidity.

**Table 1:** Descriptive characteristics of the study population.

		**Study population**		
		***N***	**Column (%)**	**Unvaccinated (%)**
**Demographic characteristics**				
**Age**	26–29	320 339	5.0%	35.9%
	30–49	2 335 371	36.5%	30.4%
	50–59	1 106 548	17.3%	21.6%
	60–69	1 086 453	17.0%	16.8%
	70+	1 550 057	24.2%	12.0%
**Sex**	Women	3 457 371	54.0%	22.0%
	Men	2 941 397	46.0%	22.9%
**Health characteristics**				
**Comorbidity index**	DCCI = 0	3 699 976	57.8%	26.5%
	DCCI = 1	1 330 947	20.8%	19.7%
	DCCI = 2	686 836	10.7%	15.6%
	DCCI = 3+	681 009	10.6%	12.5%
**Registration with GP**	Yes	6 250 412	97.7%	22.2%
	No	148 356	2.3%	31.6%
**Socioeconomic characteristics**				
**SES**	6 = highest SES	1 153 440	18.0%	13.1%
	5	1 230 947	19.2%	18.4%
	4	1 287 588	20.1%	22.3%
	3	1 341 189	21.0%	25.4%
	2	1 195 194	18.7%	29.0%
	1 = lowest SES	190 410	3.0%	42.1%
**Unemployment**	No	5 917 027	92.5%	21.1%
	Yes	481 741	7.5%	38.7%
**Total**		6 398 768	100.0%	22.4%

### Statistical methods

Descriptive analysis summarizes the subjects’ characteristics, including COVID-19 vaccination status. Binary logistic regression was used to test the relationship between vaccination status (0 = vaccinated, reference category vs. 1 = not vaccinated) and selected predictors (independent variables). Age was included as both linear and quadratic terms. Binary variables were sex (ref. women), registration with a GP (ref. yes-registration), and unemployment (ref. no). SES was included as a categorical variable with 6 categories, with the highest (best) quintile as reference. In the supplementary sensitivity test, logged income was included as a continuous variable. DCCI was defined as a categorical variable with four categories, ref. no comorbidities.

The results are presented as odds ratios (OR) with corresponding 95% confidence intervals (95% CI) in two models: first, the independent variables were adjusted for age and sex as the base model which allows assessment of the potential confounding; the second model is adjusted for comorbidity index, registration with GP, SES and unemployment, and for district of residence (to take into account the substantial geographical variation in vaccination rates). IBM SPSS Statistics 28 was used for statistical analyses.

## Results

Table 1 shows the distribution of the analytical sample according to the six selected characteristics. The overall share of people without completed COVID-19 vaccinations was 22.4%. The proportion of unvaccinated subjects decreased with increasing comorbidity index (DCCI) from 26.5% among those without any comorbidity to 12.5% among 3 or comorbidities, and it was higher among people not registered with a GP (31.6% vs. 22.2%). The group with the lowest economic status (the lowest income quintile receiving social benefits) was small (3.0%) but had the highest proportion of unvaccinated subjects (42.1%). Similarly, the relatively small group of unemployed individuals (7.5%) also recorded a high proportion of unvaccinated persons (38.7%). There were pronounced geographical differences in the prevalence of unvaccinated status, ranging from 15.3% up to 31.9% (Fig 2), with the lowest proportion of the population without vaccination being the capital city, Prague, and its surrounding areas.

Both indicators of social disadvantage were associated with unvaccinated status (Table 2). The ORs increased with worsening income-based SES; after adjusting for age and sex, the OR in the lowest income quintile receiving social benefits was 4.87 (95% CI 4.82–4.92) compared with the highest quintile (reference category). Additional adjustment reduced the OR to 3.89 (95% CI 3.84–3.93). Being unemployed was associated with OR of 1.94 (95% CI 1.93–1.95), which was reduced to 1.52 (95% CI 1.50–1.53) after further adjustment.

**Table 2:** ORs (95% CIs) for associations between COVID-19 unvaccinated status and sociodemographic and health variables.

		**OR (95% CI) adj. For age and sex**	**OR (95% CI) Fully adjusted** ^**a**^
Sex	Women (Ref.)	1	1
	Men	0.98 (0.98–0.99)	1.16 (1.15–1.16)
**Comorbidity index**	DCCI = 0 (Ref.)	1	1
	DCCI = 1	0.84 (0.83–0.84)	0.81 (0.80–0.81)
	DCCI = 2	0.77 (0.76–0.77)	0.73 (0.72–0.74)
	DCCI = 3+	0.71 (0.70–0.71)	0.67 (0.66–0.67)
**Registration with GP**	Yes (Ref.)	1	1
	No	1.32 (0.31–0.34)	1.25 (1.23–1.26)
**SES**	6 = highest (Ref.)	1	1
	5	1.51 (1.50–1.52)	1.43 (1.42–1.44)
	4	1.93 (1.92–1.94)	1.79 (1.78–1.80)
	3	2.35 (2.33–2.36)	2.12 (2.10–2.13)
	2	2.79 (2.77–2.81)	2.52 (2.51–2.54)
	1 = lowest	4.87 (4.82–4.92)	3.89 (3.84–3.93)
**Unemployment**	No (Ref.)	1	1
	Yes	1.94 (1.93–1.95)	1.52 (1.50–1.53)

a The model includes age, sex, district of residence, comorbidity index, registration with GP, SES and unemployment.

Other covariates were also significantly associated with vaccination status, although the differences among the categories were smaller than those for socioeconomic indicators. In the fully adjusted model, men had 16% higher odds of being unvaccinated (OR = 1.16; 95% CI 1.15–1.16) than women; subjects not registered with a GP had 25% higher odds of being unvaccinated (OR = 1.25; 95% CI 1.23–1.26), and the odds of remaining unvaccinated decreased with increasing values of the morbidity index. For the population with three or more registered comorbidities (DCCI = 3+), the OR of being unvaccinated was 0.67 (95% CI 0.66–0.67) lower compared with the population without any comorbidities.

The first set of sensitivity analyses, where the associations with SES were analyzed separately for the economically active (for which SES categories were derived from income) and for retirees (for which SES was derived from retirement pensions), produced very similar results in both groups (Supplementary Fig. 1). In the second sensitivity analysis, income was modeled as a continuous variable (log-transformed) with a binary indicator of social benefit receipt to capture extreme disadvantage as a distinct covariate (Supplementary Table 2). The results indicated a significant effect of income, with OR 0.31 (95% CI 0.30–0.31) per 1-point increase of log10-transformed income (corresponding to ~4.8% reduction of the odds that a person will not be vaccinated with a 10% increase in income). The fit of the model measured using Nagelkerke R2 was the same as if using categorical SES (0.90), so including income as a continuous variable did not affect the fit of the model. Overall, the results of the sensitivity analyses support the main findings in terms of direction, magnitude, and statistical significance of the associations.

## Discussion

### Main findings of this study

This study examined the predictors of COVID-19 vaccination status using linked data from several national registries in Czechia. We found that more than a fifth of adults in Czechia remained unvaccinated against COVID-19. These were more likely to be younger, of lower SES, job seekers, not registered with a GP, and without comorbidities (when adjusted for age and place of residence). The most pronounced differences were found between groups characterized by economic indicators. In particular, the most income-disadvantaged group, although small in size, had the prevalence of unvaccinated status over 40% and four-fold increased odds compared to the most affluent group, suggesting a potentially important pocket of high-risk population.

### What is already known on this topic

Studies in Western countries have consistently found a positive association between SES and COVID-19 vaccination,^1–8^ but there is much less evidence from CEE. Surveys in Estonia, Hungary, Russia, and Czechia suggested that females and younger, less educated, and unemployed subjects seem more likely to reject the vaccine. Education tends to be used as the primary indicator of SES in CEE, with limited evidence on indicators of material aspects of SES, such as income. Until now, the evidence on vaccination behavior in the Central and Eastern European region during the pandemic has been limited and mostly based on questionnaire-based surveys and self-reports.

### What this study adds

This largest individual-level study of COVID-19 vaccination using objective data in CCE to date provides robust evidence on social inequality of vaccination status. Consistent with evidence from Western country, we found an association between socioeconomic disadvantage and the odds of remaining unvaccinated. The observed effect of material disadvantage was larger in our study compared to previous studies utilizing data from national registers. While we found almost a four-fold increase in the odds of unvaccinated status among the individuals in the lowest quintile receiving social benefits, findings from Sweden,^5^ Belgium,^2^ and Switzerland^3^ suggested approximately a two-fold increase in odds of not receiving the vaccine in the lowest SES group. The effect of unemployment in the fully adjusted model was similar to the effect reported by Spetz *et al.*^5^ and Cavillot *et al.*,^2^ with the OR of ~1.5.

One of the contributions of this study was the quantification of socioeconomic and health inequalities in vaccination use. Socioeconomic inequalities in COVID-19 vaccine uptake persisted after controlling for age, sex, access to primary care, and health status. Inequalities in SES in COVID-19 vaccine uptake may be linked to levels of health literacy and levels of trust in political elites and decisions.^16^^,^^17^ Another socioeconomic risk factor for lower vaccination rates we explored was individual-level unemployment. This variable may reflect marginalization, lower levels of awareness, and social pressure to vaccinate.^18^ Unlike employees motivated by their employer to be vaccinated during the pandemic (e.g. those vaccinated were not required to be tested), the unemployed were often not subjected to similar requirements. Additionally, the unemployed persons often do not have the same opportunities or access to important resources and services. As a result, vaccination was not an immediate necessity for them.

As in previous studies,^1^^,^^4^^,^^5^^,^^7^ younger individuals were less likely to receive the vaccine than older individuals. Although some studies found that women were less likely to get vaccinated than men,^7^^,^^19^^,^^20^ our results suggested lower COVID-19 vaccine uptake among men, similar to that reported by Cavillot *et al.*^2^ and Spetz *et al.*^5^ We found that the lowest proportion of the population without vaccination resided in the capital city of Prague and its surrounding areas. As previous studies repeatedly reported lower vaccination rates in socioeconomically deprived areas,^1^^,^^15^^,^^21–23^ this finding may be explained by the area’s socioeconomic advantage, which is among the highest in the Czechia.

Similarly to previous reports,^1^^,^^3^ individuals with fewer comorbidities were more likely to receive the vaccination, which may reflect the willingness to be vaccinated. Individuals with higher disease severity were vaccinated more often, which is consistent with findings from the UK and the USA.^24^^,^^25^ In general, individuals at higher risk of ill health related to COVID-19 may have been more motivated and perhaps more likely to be prioritized to be vaccinated. Finally, people not registered with a GP were more often unvaccinated. It can be speculated that this may reflect negative attitudes and/or distrust in vaccination, the health system, and the government system in general. The use of primary health care is associated with better preventive health behaviors, including vaccination.^26^ Specific targeting of vaccination campaigns at vulnerable and marginalized groups of people could increase the effectiveness of these campaigns in future pandemics and contribute to reducing socioeconomic inequalities in COVID-19 vaccine uptake.^17^

### Limitations of this study

Several limitations of this study should be considered. First, about 16% of potentially eligible subjects were excluded due to missing data, mainly on income. The prevalence of unvaccinated status in excluded subjects was almost twice of that in included subjects, and the excluded groups also differed in terms of demographic structure. In addition, the analyses did not include persons who died during the pandemic. Given the limited number of variables in these administrative data, missing values (particularly income) could not be reliably imputed. These issues limit the extrapolation of the finding to the whole population. Second, the lack of data on education and occupational characteristics may have overemphasized the role of material conditions (income) while missing the contribution of education-related influences. On the other hand, obtaining reliable income data using surveys faces complications such as inaccurate data and high non-response.^27^ The use of administrative data partly addresses these limitations, although some misclassification of income and material deprivation is likely. Unemployment serves as an indicator of reduced employment stability alongside SES.

An important limitation of the income-based SES indicator is the way it is constructed. While using the percentile distribution of income from employment and from pensions allowed us to include both economically active individuals and retirees in the model, it may obscure absolute income variation, especially at the extremes, making socioeconomic gradients harder to detect. Grouping incomes into quintiles relies on arbitrary cutoffs, potentially biasing estimates. Pension-based percentiles may also poorly reflect pre-retirement earnings. However, both sets of sensitivity analyses (i.e. separate analysis of economically active individuals vs. retirees and analyzing income as a continuous variable) support the robustness of our findings.

Third, there was a considerable geographical variation in vaccination uptake; our analysis controlled for the district of residence, but the geographical context will require further analysis. Finally, given the nature of the data, there is no precise temporal sequence of events or changes during the analyzed period (e.g. whether receiving social benefits occurred before vs. after the decision to undergo vaccination, etc.).

## Conclusions

The COVID-19 pandemic uncovered the inequities relating to access and acceptance of vaccines within and across countries. SES and access to primary care highlight structural inequalities that influence vaccination willingness. Addressing the needs of the vulnerable and marginalized groups through evidence-based research using the national register data is essential in improving vaccine uptake and acceptance of preventive measures in future crises.

## Supplementary Material

COVID_Supplementary_material_2nd_revision_clean_fdag043

## Data Availability

Only National Health Information System (NHIS) is allowed to access the raw data from national register. Due to legal restrictions, the data cannot be accessed externally.

## Notes

**Dagmar Dzúrová**, Professor of Demography

**Klára Benešová**, Researcher, Data Scientist

**Josef Bernard**, Associate Professor of Sociology

**Klára Hulíková Tesárková**, Associate Professor of Demography

**Jiří Jarkovský**, Researcher, Data Scientist

**Lukáš Kahoun**, Data Analyst

**Gabriela Kšiňanová**, Psychologist

**Jan Mužík**, Researcher, Data Scientist

**Pavlína Netrdová**, Associate Professor of Social Geography and Regional Development

**Daniel Szabo**, Data Scientist

**Martin Bobák**, Professor of Epidemiology
