# Supplementary material for: Predictors of COVID-19 vaccination status: a nationwide linkage study in Czechia
Source: J Public Health (Oxf). 2026 Jun 16;48(3):644–51. doi: 10.1093/pubmed/fdag043 (PMC13531712; doi:10.1093/pubmed/fdag043)
Supplement: Supplementary file 1 [file covid_supplementary_material_2nd_revision_clean_fdag043.docx]

**Supplementary material**

**Supplementary Table 1.**

**Descriptive characteristics of the analytical sample vs. persons excluded due to missing values.**

|  |  | Analytical sample | | | Persons excluded due to missing values | | |
| --- | --- | --- | --- | --- | --- | --- | --- |
|  |  | N | (%) | Unvaccinated % | N | (%) | Unvaccinated % |
| Demographic characteristics | | | | | | | |
| Age | 26-29 | 320,339 | 5.0% | 35.9% | 83,901 | 6.9% | 46.4% |
|  | 30-49 | 2,335,371 | 36.5% | 30.4% | 649,484 | 53.6% | 42.7% |
|  | 50-59 | 1,106,548 | 17.3% | 21.6% | 306,738 | 25.3% | 35.3% |
|  | 60-69 | 1,086,453 | 17.0% | 16.8% | 130,611 | 10.8% | 33.2% |
|  | 70+ | 1,550,057 | 24.2% | 12.0% | 41,259 | 3.4% | 27.3% |
| Sex | Women | 3,457,371 | 54.0% | 22.0% | 473,895 | 39.1% | 42.7% |
|  | Men | 2,941,397 | 46.0% | 22.9% | 738,098 | 60.9% | 37.5% |
| Health characteristics | | | | | | | |
| Morbidity index | DCCI=0 | 3,699,976 | 57.8% | 26.5% | 863,824 | 71.3% | 43.0% |
|  | DCCI=1 | 1,330,947 | 20.8% | 19.7% | 196,279 | 16.2% | 33.2% |
|  | DCCI=2 | 686,836 | 10.7% | 15.6% | 82,856 | 6.8% | 30.0% |
|  | DCCI=3+ | 681,009 | 10.6% | 12.5% | 69,034 | 5.7% | 26.0% |
|  | Missing | - | - | - |  |  |  |
| Registration with GP | Yes | 6,250,412 | 97.7% | 22.2% | 1,023,685 | 84.5% | 38.3% |
|  | No | 148,356 | 2.3% | 31.6% | 188,308 | 15.5% | 46.2% |
| Socioeconomic characteristics | | | | | | | |
| Socioeconomic status | 6=highest SES | 1,153,440 | 18.0% | 13.1% | 199 | 0.0% | 98.5% |
|  | 5 | 1,230,947 | 19.2% | 18.4% | 298 | 0.0% | 99.3% |
|  | 4 | 1,287,588 | 20.1% | 22.3% | 421 | 0.0% | 99.3% |
|  | 3 | 1,341,189 | 21.0% | 25.4% | 571 | 0.0% | 99.6% |
|  | 2 | 1,195,194 | 18.7% | 29.0% | 907 | 0.1% | 99.6% |
|  | 1=lowest SES | 190,410 | 3.0% | 42.1% | 109 | 0.0% | 97.2% |
|  | Missing | - | - | - | 1,209,488 | 99.8% | 39.4% |
| Unemployment | No | 5,917,027 | 92.5% | 21.1% | 1,092,766 | 90.2% | 38.1% |
|  | Yes | 481,741 | 7.5% | 38.7% | 119,227 | 9.8% | 52.9% |
| Total |  | 6,398,768 | 100.0% | 22.4% | 1,211,993 | 100.0% | 39.6% |

**Supplementary Table 2.**

**Odds ratios (95% confidence intervals) for associations between COVID-19 unvaccinated status and sociodemographic and health variables, using income(log10) as a continuous variable.**

|  | | OR (95% CI)  Fully adjusted* |
| --- | --- | --- |
| Sex | Women (Ref.) | 1 |
|  | Men | 1.17 (1.17-1.18) |
| Comorbidity index | DCCI=0 (Ref.) | 1 |
|  | DCCI=1 | 0.83 (0.83-0.84) |
|  | DCCI=2 | 0.76 (0.76-0.77) |
|  | DCCI=3+ | 0.67 (0.66-0.68) |
| Registration with GP | Yes (Ref.) | 1 |
|  | No | 1.18 (1.17-1.20) |
| Income (log 10) |  | 0.31 (0.30-0.31) |
| Any social benefit | Yes (Ref.) | 1 |
|  | No | 0.70 (0.69-0.70) |
| Unemployment | No (Ref.) | 1 |
|  | Yes | 1.51 (1.50-1.52) |

* The model includes age, sex, district of residence, comorbidity index, registration with GP, socioeconomic status and unemployment.

**Supplementary Figure 1.**

**Odds ratios of unvaccinated status by SES category relative to "highest SES" for economically active and retirees separately. SES is categorised into 6 categories.**

|  | Total | Economically active | Retirees |
| --- | --- | --- | --- |
| Lowest SES | 3.88822 | 3.8602 | 4.21748 |
|  | 2.52417 | 2.53577 | 2.67714 |
|  | 2.11576 | 2.24564 | 1.88885 |
|  | 1.78731 | 1.91979 | 1.54233 |
| Second highest SES | 1.43052 | 1.527 | 1.27477 |
